# Mixed Spatial and Movement Representations in the Primate Posterior Parietal Cortex

**DOI:** 10.3389/fncir.2019.00015

**Published:** 2019-03-11

**Authors:** Kostas Hadjidimitrakis, Sophia Bakola, Yan T. Wong, Maureen A. Hagan

**Affiliations:** ^1^Department of Physiology, Monash University, Clayton, VIC, Australia; ^2^Australian Research Council Centre of Excellence for Integrative Brain Function, Monash University Node, Clayton, VIC, Australia; ^3^Department of Electrical and Computer Science Engineering, Monash University, Clayton, VIC, Australia

**Keywords:** eye movements, reaching, grasping, PPC, posterior parietal cortex, movement planning

## Abstract

The posterior parietal cortex (PPC) of humans and non-human primates plays a key role in the sensory and motor transformations required to guide motor actions to objects of interest in the environment. Despite decades of research, the anatomical and functional organization of this region is still a matter of contention. It is generally accepted that specialized parietal subregions and their functional counterparts in the frontal cortex participate in distinct segregated networks related to eye, arm and hand movements. However, experimental evidence obtained primarily from single neuron recording studies in non-human primates has demonstrated a rich mixing of signals processed by parietal neurons, calling into question ideas for a strict functional specialization. Here, we present a brief account of this line of research together with the basic trends in the anatomical connectivity patterns of the parietal subregions. We review, the evidence related to the functional communication between subregions of the PPC and describe progress towards using parietal neuron activity in neuroprosthetic applications. Recent literature suggests a role for the PPC not as a constellation of specialized functional subdomains, but as a dynamic network of sensorimotor loci that combine multiple signals and work in concert to guide motor behavior.

## Introduction

Humans and non-human primates make skillful reaching-to-grasping movements that are tightly coordinated in space and time (Jeannerod et al., 1995). Moreover, eye movements often accompany every day actions towards objects, supplying information about object identity and location, and guiding arm movements (Johansson et al., 2001; Land and Hayhoe, 2001; Hayhoe et al., 2003). Contemporary research has established that the posterior parietal cortex (PPC) is involved in the representation of spatial information and goal-directed behavior using different motor effectors (Husain and Nachev, 2007; Andersen and Cui, 2009). Since the original unified view of PPC as a “command apparatus for the operation of the limbs, hands and eyes” (Mountcastle et al., 1975), anatomical, neurophysiological and neuroimaging evidence has ascribed the neural encoding of looking, reaching and grasping actions to distinct PPC sectors (Rizzolatti and Matelli, 2003; Vesia and Crawford, 2012; Andersen et al., 2014).

At the same time, numerous studies have shown convergence of eye-, arm- and/or hand-related signals, both within single PPC sectors and at the level of individual cells, although which of these signals play a casual role in defining functional specificity would require future investigations. Recent research findings raise several issues regarding the potential substrates of distinct movements in parietal cortex and the information flow between the various PPC sectors. Here, we outline evidence, mainly from non-human primate anatomical and neurophysiological studies, for the rich variety of signals carried by PPC neurons related to movement guidance that suggests a more widespread representation of movement variables than previously assumed. From a clinical perspective, the diverse representation of signals from parietal cortex may prove useful for the design of more efficient neuroprosthetic devices for patients who cannot reach and grasp objects either because of loss of arms or lesions of the motor pathways.

## Anatomical Organization of the Posterior Parietal Cortex

**Figure 1:**
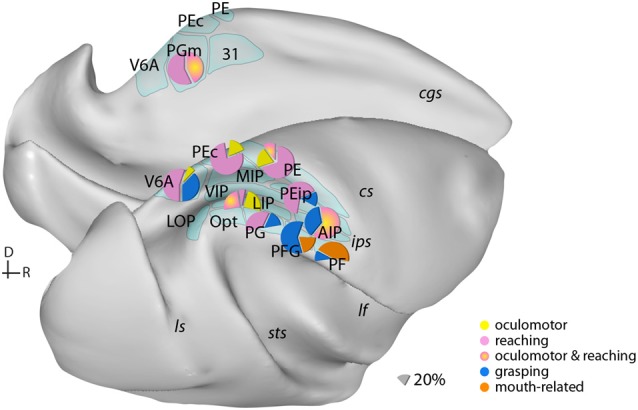
Examples of the distribution of multiple movement preparatory/execution signals related to different effectors in posterior parietal areas. Only the most frequent movement types are illustrated. Mouth-related actions refer mainly to biting. Data derived from electrophysiological studies: Ferraina et al. (1997) (PGm); Battaglia-Mayer et al. (2001) (PEc); Dickinson et al. (2003) (LIP); Kutz et al. (2003); Fattori et al. (2005, 2010) (V6A); Gardner et al. (2007) (PEip); Rozzi et al. (2008) (PG, PFG, PF); Archambault et al. (2009) (PE). Lateral (bottom) and medial (top) views of a macaque brain show our current knowledge of the anatomical organization of posterior parietal cortex (PPC). Abbreviations of sulci: cgs, cingulate; cs, central; ips, intraparietal; lf, lateral fissure; ls, luneate; sts, superior temporal. D, dorsal; R, rostral.

The PPC is composed of several areas that vary in histological features and connections with other parts of the brain. Definitions of areas have evolved over time from the historical assignment of posterior parietal fields to areas 5 and 7 of Brodmann to more refined schemes (e.g., Figure 1) but, despite general consensus on the number and characteristics of individual areas, maps produced by different groups vary widely and functional subdivisions do not always appear to respect architectonic boundaries (e.g., Savaki et al., 2010; Arcaro et al., 2011; Seelke et al., 2012). Nonetheless, in non-human primates, the anatomical organization of PPC is shaped by the relative influence of sensorimotor input to different areas. Segregated projections from the motor control centers in the frontal lobe are distributed along the dorsal-ventral extent of PPC. Primary motor cortex connects mainly to the parietal convexity (PE) and rostral parts of the medial bank of the intraparietal sulcus (IPS; PEip). Caudal superior and medial parietal areas (V6A, MIP, PEc, 31) connect preferentially with parts of dorsal premotor cortex, whereas inferior parietal areas (PFG, PF, AIP, VIP) connect with the ventral premotor cortex (Marconi et al., 2001; Tanné-Gariépy et al., 2002; Rozzi et al., 2006; Borra et al., 2008; Gamberini et al., 2009; Bakola et al., 2010, 2017; Passarelli et al., 2011, 2018). Input to LIP (Blatt et al., 1990; Lewis and Van Essen, 2000) and PGm (Cavada and Goldman-Rakic, 1989; Passarelli et al., 2018) originates mainly in the oculomotor-related frontal eye fields (FEFs). Segregation of motor projections is not in absolute terms, though, since each parietal area usually receives convergent input from other structures; e.g., PEip receives additional projections from ventral premotor cortex (Tanné-Gariépy et al., 2002; Bakola et al., 2017).

A relative segregation of sensory-specific projections has been described along the rostral-caudal dimension, with somatic-related input targeting heavily rostral parietal areas (Rozzi et al., 2006; Bakola et al., 2013; Padberg et al., 2019). Visual inputs (in particular representations of peripheral vision) are prominent in caudal parietal areas, however there is variation in the source of visual afferents to PPC. For example, numerous afferents to V6A (Passarelli et al., 2011) and LIP (Lewis and Van Essen, 2000) originate in area V6, whereas caudal inferior parietal lobe receives almost exclusively projections from the motion area MST of the temporal cortex (Rozzi et al., 2006). Several projections to MIP and PGm arrive also from the putative visual region (Kobayashi and Amaral, 2003), ventral to PGm (Bakola et al., 2017; Passarelli et al., 2018). In addition to sensorimotor input, PPC receives segregated input from other systems. For example, caudal/medial areas receive projections from limbic fields of the brain (Rozzi et al., 2006; Bakola et al., 2017; Passarelli et al., 2018). These include projections from the posterior cingulate and retrosplenial regions and area prostriata (Yu et al., 2012) and likely represent routes by which information about spatial orientation and memory reaches parts of PPC (Vann et al., 2009; Kravitz et al., 2011).

Despite the diversity of extrinsic connections, short-range intrinsic connections between adjacent parietal areas form a substantial component of areal connectivity, highlighting the potentially large influence of local processing in defining the function of PPC sectors (Caminiti et al., 2017). This organization may support synergistic actions of different effectors to produce meaningful movements (Kaas and Stepniewska, 2016; Catani et al., 2017).

## Functional Response Properties in Individual Regions of the Posterior Parietal Cortex

Two exemplar nodes of the functional specialization view on PPC are areas AIP and LIP that have been associated with the control of hand-object interactions required for grasping and for the guidance of eye movements, respectively (Gallese et al., 1994; Andersen et al., 1998; Murata et al., 2000; Cui and Andersen, 2007). By comparison, planning and execution of reaching movements appear to be distributed in several areas of the superior (V6A, PEc, MIP and PE/PEip) and inferior parietal lobe (Snyder et al., 1997; Battaglia-Mayer et al., 2000, 2007; Fattori et al., 2005; Heider et al., 2010; McGuire and Sabes, 2011; Hadjidimitrakis et al., 2012, 2015).

Influential models for parallel parietal-frontal networks for motor actions have dominated parietal research in the past (Jeannerod et al., 1995; Matelli and Luppino, 2001). Accordingly, reach-related signals flow from the superior parietal to the dorsal premotor cortex and grasp-related activity is conveyed from AIP to ventral premotor cortex; both streams converge to the primary motor cortex (Burman et al., 2014; Dea et al., 2016). Re-evaluation of these models became necessary after studies showing that individual premotor neurons carried both reaching and grasping information (Raos et al., 2004; Stark et al., 2007). Along these lines, later work reported grasping parameters to be coded in the traditionally reaching domains of the superior parietal cortex (Chen et al., 2009; Fattori et al., 2010). Furthermore, single AIP neurons encoded both the reaching direction and grip type (Lehmann and Scherberger, 2013).

Additional evidence for the mixing of neural signals comes from work on the spatial reference frames used for reaching movements. Until recently, the dominant view was that neurons in each parietal area have uniform reference frames. A serial organization of reach-related responses along the extent of PPC has been reported, with responses coding target locations relative to the eyes (eye-centered frame) recorded caudally and responses coding locations in head-, body- and hand-centered frame rostrally (Flanders et al., 1992). This view found support in studies that showed eye-centered reference frames caudally in the parietal reach region (PRR, Snyder et al., 1997) and hand-centered representations rostrally in area PE (Lacquaniti et al., 1995; Batista et al., 1999; Buneo et al., 2002; Marzocchi et al., 2008). However, later work showed that neurons in single PPC areas encode reaches relative to the eye, hand, head and body (Mullette-Gillman et al., 2009; Chang and Snyder, 2010; McGuire and Sabes, 2011; Hadjidimitrakis et al., 2014b; Bosco et al., 2016; Piserchia et al., 2017). The presence of mixed, eye- and limb-centered, reference frames within several PPC areas challenges the one-to-one association of a particular type of reference frame with one region and, subsequently, the view of serial reference frame transformations across the PPC “reach” network (McGuire and Sabes, 2011).

Mixing of signals has also been observed at another level of movement control. The distance and direction of reach goals, which were considered to have independent neuronal substrates (Crawford et al., 2011), were encoded by largely overlapping neuronal populations in V6A and PEc (Hadjidimitrakis et al., 2014a, 2015; Filippini et al., 2018). Furthermore, PRR neurons can simultaneously encode multiple potential movement goals (Baldauf et al., 2008; Klaes et al., 2011), thus further illustrating the richness of the selectivity.

In a recent human study, Zhang et al. (2017) reported a mixture of effector representations in populations of neurons in the putative homolog of macaque AIP, arguing against a strict anatomical segregation of body parts. Using fMRI repetition suppression, Heed et al. (2016) examined activity in the PPC in humans performing delayed eye, hand and foot movements to visual targets. They reported a gradient of organization schemes along the extent of PPC, with a region activated independently of the effector used among regions showing effector specificity. Accordingly, the view that emerges is that the primate PPC hosts multiple representations of motor actions, with individual areas and networks (e.g., reaching network) showing only a relative emphasis on a particular effector or movement type.

## A Potential Network for Eye-Arm Coordination

The mixed selectivity and overlapping representations for different movements in PPC make it an ideal site for mediating complex behaviors like eye-hand coordination. Indeed, growing evidence suggests that coordinated behaviors, such as eye-hand movements, rely on parietal circuits. Reaction times for eye and hand movements are correlated (Dean et al., 2011), suggesting a common neural mechanism. The mixing of various types of signals in single PPC neurons and sectors could be interpreted as a manifestation of coordinated activity. For example, most LIP neurons fire stronger when a combined reach and saccade is planned compared to a saccade alone (Hagan et al., 2012). Neural correlates for single and combined eye- and arm-related movements were reported in several PPC fields (Battaglia-Mayer et al., 2001; Calton et al., 2002; Dickinson et al., 2003), with activity being usually weaker for the non-preferred movement. Moreover, neural responses are modulated by static eye and arm position in PEc, V6A and the caudal inferior parietal lobe (Battaglia-Mayer et al., 2000, 2007; Breveglieri et al., 2012, 2014; Piserchia et al., 2017).

The mixing of signals within PPC may result from the short-range intrinsic connections between adjacent parietal areas (Caminiti et al., 2017). In order to understand the mixed selectivity and how it relates to complex behaviors, simultaneous recordings from multiple PPC areas are necessary. However, very few works have employed this method in PPC (e.g., Cui and Andersen, 2007; Dean et al., 2012). By comparison, increasingly interactions between areas of the frontal and parietal cortex are being studied. Multi-area recordings in primates allow for correlations between the activity across areas to be studied and have complemented non-invasive work using fMRI and MEG.

In electrophysiological studies, the local field potential (LFP) has been instrumental in understanding the relationship in neural activity across brain areas. The LFP is composed of synaptic and spiking activity in the vicinity of the recording electrode (Mitzdorf, 1985), and gives an estimate of the population activity. Like spiking-activity, the LFP power is tuned to saccade and reach direction in LIP and PRR, respectively (Pesaran et al., 2002; Scherberger et al., 2005). Synchrony, or coherence, between the firing rates of individual neurons and the LFP at different frequencies may reflect the processing of different types of information (Fries, 2005). During coordinated eye-hand movements, the beta-band (~15–30 Hz) LFP activity decreases around movement initiation in both LIP and PRR, and correlates with the reaction times for coordinated reach and saccades (but not for saccades made alone, Dean et al., 2012). Furthermore, LIP neurons with reduced activity during eye-hand movements, compared to saccades, tend to be coherent with the beta-band LFP (Hagan et al., 2012) and their firing rate predicts the reaction times of coordinated eye-hand movements. This suggests that these neurons participate in a neural circuit that orchestrates coordinated eye-hand movements (Dean et al., 2012). Coherent activity across areas may also contribute to the processing of cognitive signals such as decision-making (Hawellek et al., 2016; Wong et al., 2016) and visual attention in PPC (Buschman and Miller, 2007; Saalmann et al., 2007; Gregoriou et al., 2009).

The studies of LFP-firing coherence are limited in their ability to provide causal evidence of the role of the PPC in eye-hand coordination. In this regard, a number of inactivation studies in PPC have provided more direct evidence, with two works reporting effects on limb (but not eye) movements (Hwang et al., 2012; Yttri et al., 2014), whereas another one found disrupted eye-hand correlations after bilateral inactivation (Battaglia-Mayer et al., 2013). Furthermore, unilateral inactivation of LIP combined with fMRI resulted in rapid spatial reorganization in the active hemisphere (Wilke et al., 2012), suggesting that the functions of PPC are likely spread over a wider network that extends over both hemispheres. This could also explain recent evidence showing no effect of unilateral LIP inactivation on decision-making (Katz et al., 2016). Similarly, inactivation of VIP had no effect on behavior in a heading discrimination task (Chen et al., 2016). In humans, fMRI-guided transcranial magnetic stimulation demonstrated a causal role of the anterior portion of the IPS to reaching (Reichenbach et al., 2011). Overall, inactivation evidence should be treated cautiously. More sensitive activity manipulations could be useful to determine how PPC nodes contribute to motor behaviors. The use of sophisticated tools such as optogenetics in primates (Jazayeri et al., 2012; Watakabe et al., 2016; El-Shamayleh et al., 2017) could help overcome current limitations.

## Implications of Mixed Selectivity in the PPC for Medical Interventions

The diversity of signals within the PPC has sparked great interest to the neuroprosthetic community. For patients suffering from loss of function due to paralysis or amputation of a limb, there can be great difficulty in interacting with people or everyday objects. Brain machine interfaces (BMIs) offer some hope in helping remedy these difficulties. A BMI is a device that can record neural activity from the brain while subjects think about a certain task, and then *via* a decoder, extract the subject’s intentions. These decoded intentions are used to control external devices that can vary from a cursor on a monitor, to an anthromorphic robotic arm and hand, to a functional electrical stimulator to activate paralyzed muscles.

Most commonly, electrodes are implanted in the primary motor and premotor areas while patients use motor imagery to provide the necessary input to these BMIs (Markowitz et al., 2011; Hochberg et al., 2012; Collinger et al., 2013). Devices implanted in the motor areas typically decode the trajectory of an effector. Early studies showed that PPC neurons could be used in conjunction with frontal motor areas to control closed loop BMIs, however it was unclear to what extent the PPC neurons contributed to the efficacy of these devices (Wessberg et al., 2000). In a study that compared offline decoding of hand position and velocity in non-human primates, decoding with PPC neurons was inferior to the decoding performance achieved with primary motor and dorsal premotor cortex (Carmena et al., 2003), possibly indicating that the PPC neurons were not contributing much to the overall control.

However, Musallam et al. (2004) went on to demonstrate that high level movement goal information as well as expected reward values of different targets could be decoded from signals in PRR to control a cursor on the screen during a BMI task. These control signals could be generated in the absence of an actual movement. The goal signals allow an abstraction away from the low-level commands necessary to achieve the wanted action as well as the device that actually enacts the action. These low-level commands can be generated through external optimal control algorithms. Goals for multiple sequential movements are planned in PRR (Baldauf et al., 2008) but not in the superior parietal convexity (Li and Cui, 2013) providing a rich mix of signals.

However, soon after this, trajectory information was successfully decoded from the medial bank of the IPS as well as the dorsal convexity to allow control of a 2-dimensional (2D; Mulliken et al., 2008a,b) as well as 3D (Hauschild et al., 2012) cursor on a screen. Decoding algorithms to incorporate the cognitive neural signals and the trajectory information will also provide increased performance compared to each type of signal alone (Shanechi et al., 2013a,b). These studies primarily focused on decoding of spiking activity, but similar information could be extracted from the LFP (Andersen et al., 2004; Scherberger et al., 2005).

The clinical relevance of the PPC to neural prosthetics was demonstrated in the first human trial of a BMI that utilized neural signals from the PPC (Aflalo et al., 2015). In this study, a tetraplegic patient was implanted with electrode arrays in putative areas 5d/PE and AIP and could successfully control 2D and 3D cursors as well as a robotic limb. Therefore, exploiting the richness of information in the PPC may be an advantageous strategy for developing more efficient BMIs.

## Concluding Remarks

Despite decades of research, a definitive understanding of how individual brains areas are defined, perform distinct computations, and interact with other brain areas remains elusive. The PPC has proved an ideal test bed for understanding how the underlying neural architecture supports a range of sensory, motor and cognitive functions. Anatomy and physiology provide distinct lines of evidence for characterizing the brain areas of the PPC less as a cluster of finite regions and more as a network of integrated areas that may flexibly form the neural basis for diverse functions. The future of systems neuroscience is in understanding how these brain areas work in concert with one another and how the neural dynamics can be used for powering the next generation of prosthetic devices.

## Author Contributions

KH, SB, YW and MH contributed to the preparation, writing and revising of this text.

## Conflict of Interest Statement

The authors declare that the research was conducted in the absence of any commercial or financial relationships that could be construed as a potential conflict of interest.
